# Smart Adhesive Joint with High-Definition Fiber-Optic Sensing for Automotive Applications

**DOI:** 10.3390/s20030614

**Published:** 2020-01-22

**Authors:** Stephen Young, Dayakar Penumadu, Darren Foster, Hannah Maeser, Bharati Balijepalli, Jason Reese, Dave Bank, Jeff Dahl, Patrick Blanchard

**Affiliations:** 1 Tickle College of Engineering, the University of Tennessee, Knoxville, TN 37996, USA; dpenumad@utk.edu (D.P.);; 2 The Dow Chemical Company, Midlands, MI 48667, USA; 3 Ford Research & Innovation Center, Dearborn, MI 48124, USA

**Keywords:** fiber-optic sensing, adhesive, joining, smart-joint, distributed, on-demand, structural health monitoring

## Abstract

Structural health monitoring of fiber-reinforced composite-based joints for automotive applications during their manufacturing and on-demand assessment for its durability in working environments is critically needed. High-definition fiber-optic sensing is an effective method to measure internal strain/stress development using minimally invasive continuous sensors. The sensing fiber diameters are in the same order of magnitude when compared to reinforcement (glass, basalt, or carbon fibers) used in polymer composites. They also offer a unique ability to monitor the evolution of residual stresses after repeated thermal exposure with varying temperatures for automotive components/joints during painting using an electrophoretic painting process. In this paper, a high-definition fiber-optic sensor utilizing Rayleigh scattering is embedded within an adhesive joint between a carbon fiber-reinforced thermoset composite panel and an aluminum panel to measure spatially resolved strain development, residual strain, and thermal expansion properties during the electrophoretic paint process-simulated conditions. The strain measured by the continuous fiber-optic sensor was compared with an alternate technique using thermal digital image correlation. The fiber-optic sensor was able to identify the spatial variation of residual strains for a discontinuous carbon fiber-reinforced composite with varying local fiber orientations and resin content.

## 1. Introduction

Carbon fiber–reinforced composites (CFRCs) are attractive to the automotive industry due to the growing need for lightweight applications to improve performance and fuel efficiency and reduce carbon emissions. Chopped fiber CFRC sheet-molded composites (SMCs) offer several advantages in producing near-net shaped complex automotive parts using compression molding techniques. Resulting material properties can be tuned for high strength, low weight, resistance to corrosion, and the potential for high-volume production as an alternative to aluminum (Al)- and magnesium (Mg)-based alloys; however, the cost of raw materials remains a persistent barrier to market entry for practical large-scale production and complex parts such as lift-gates or deck-lids require multiple-part stamping and assembly [1,2,3,4]. Thus, SMC-based materials with their improved mechanical properties and processing characteristics for flow during molding, show a lot of promise.

The mechanical integrity and thermal stability of manufactured SMC parts are largely dependent on critical parameters, including fiber orientation, voids, fiber volume fraction, and thermal response. Hybrid materials, created by joining composites and metals using suitable adhesives, have shown promise as alternatives to mechanical fastening parts, offering several advantages, including high strength, high fatigue life, and better stress distribution [5,6,7]. It is well known that adhesive bonding offers several advantages, including welding, but due to reduced stress concentrations without suitable structural health monitoring (SHM), the integrity of adhesive joints is difficult to inspect [8]. In addition, a coefficient of thermal expansion (CTE) mismatch can exist between the composite, adhesive bond, and metal substrates in hybrid joints resulting in large thermally induced stresses during the adhesive curing cycle and subsequent painting process. The local residual strains and its spatial variation are reported to have impact on material processing, strength, and ductility [4,9,10]. For large-volume materials, it is critical to monitor the processing conditions and thermal properties of the final composite part using effective methods to assess quickly the quality of the part that is required [11]. The same holds true for adhesively joined parts utilizing composite-to-composite joints or composite-to-metal hybrid joints.

In-situ monitoring of the curing cycle has gained interest for its applications in the infusion and curing of composite laminates [12,13]. SHM using optical frequency-domain reflectometry (OFDR) fiber-optic sensing and digital imaging has been applied to infrastructure applications and wind-turbine blade manufacturing, but there is a need to better understand its use in smart joints for automotive applications [14,15]. Fiber Bragg grating (FBG) has been used for monitoring strain, essentially in optical fibers with a discrete sensing zone determined a priori and located along the fiber in response to an applied strain, to obtain strain distribution in regions of interest [4,16]. In recent years, high-definition fiber optics (HD-FOS) using OFDR has been utilized as an alternative to FBG, providing the unique ability to measure strain without the need for etched gratings along the fiber. HD-FOS possesses unique key advantages including the use of standard communications-grade optical fiber that is lightweight, immune to electrical interference, has a small gage length, is abundantly available at low cost, and offers a high spatial resolution. Additionally, HD-FOS has sufficient sensitivity using Rayleigh scattering to be able to be embedded in composites during its manufacturing (Rayleigh scattering is more advantageous than Raman and Brillouin scattering, which both have weaker scattering responses). All these factors make HD-FOS suitable for measuring strain and temperature, and show its promise for the inline manufacturing and curing of parts. Each optical fiber has a distinct “fingerprint,” along the fiber that corresponds to the position of the strain, due to the molecular arrangement of the glass fiber [12,17,18,19].

In this study, the authors investigated the strain development of adhesive hybrid joints that use a thermoset adhesive to bond a carbon fiber–reinforced SMC (CFSMC) composite panel to an aluminum panel. The parameters influencing curing, such as the modulus of the adhesive, were considered. In addition, the authors implemented a new technique that correlates digital imaging with optical cameras, as well as temperature data from a thermal camera, for validation of fiber-optic sensor–based results. The benefit of thermal digital image correlation (TDIC) is its ability to quickly obtain the thermal mapping of the composite to evaluate the local fiber orientation and resin-rich areas of the surface. This technique affords the ability to inspect a large area of the part to obtain a high spatial local orientation and obtain anisotropic regions [20,21]. In this paper, the use of HD-FOS to monitor the evolution of internal and residual strains is demonstrated with high spatial and temporal resolution, as well as on-demand sensing capability using the data acquisition rate (1–100 Hz) and two types of adhesives during and after manufacturing of the joint. Due to material manufacturing methods and joining processes, there is a pressing need to develop experimental techniques that provide spatially resolved strains from thermal loading, hygroscopic concentration changes, and material interfaces in composites. This paper introduces for the first time the use of high-definition fiber-optic sensing for addressing this important need in automotive and infrastructure application space.

## 2. Sensing Principle of the High-Definition Fiber-Optic Sensor (HD-FOS)

Two commercial optical distributed–sensor interrogators acquired from LUNA (Blacksburg, VA), a multichannel unit (ODiSI 6100) and a single-channel unit (ODiSI-B), were used to measure the strain on CFSMC panels and CFSMC/Al smart joints, respectively.

**Figure 1:**
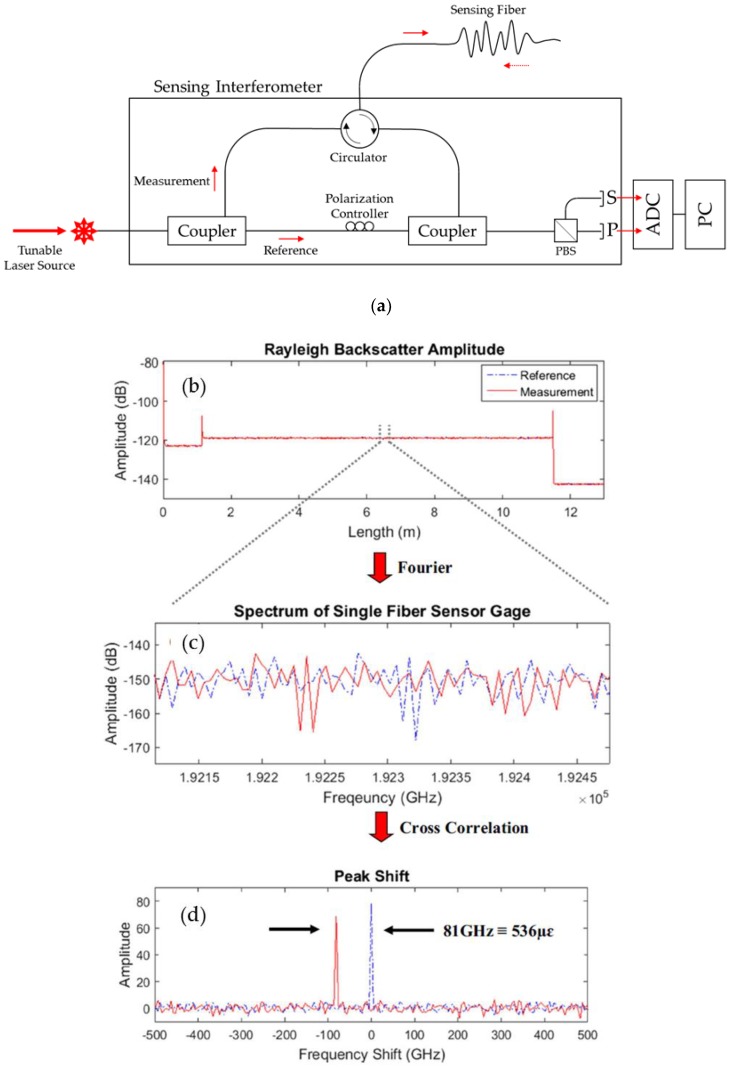
(**a**) Schematic of basic principle of optical frequency domain recorder (OFDR). (**b**) Example signature Rayleigh scatter measurement with reference and measured strain (from external stimuli) values, where (**c**) an inverse Fourier transform is applied from the gage length showing (**d**) a cross-correlation of the two spectra resulting in peak shift between the reference measurement and measured strain, after [18].

Both interrogators utilize swept-wave interferometry and can detect numerous measurements over the length of the optical fiber, with gauge pitch intervals as low as 0.64 mm coupled with data acquisition rates up to 100 Hz [22,23]. The principles of OFDR and applications of OFDR to the LUNA interrogators have been described in detail elsewhere [15,17,19,24,25]. Briefly, Figure 1 shows a schematic of the measurement principle and an example of the Rayleigh backscatter signature along the HD-FOS. The swept-wavelength interferometer measures the Rayleigh backscatter (RBS) from induced random fluctuations along the index of refraction in the fiber core. This RBS is measured as a function of the position of the fiber where the amplitude of light scattered produces a repeatable profile that is unique and static property or “fingerprint” of the optical fiber. The baseline or tared measurements (corresponding to a reference state state) are compared to measured strain resulting from an external stimuli of either applied mechanical stress or temperature. The basic configuration of a fiber-optic interrogator includes a tunable laser source (TLS), which is split between the reference and measurement arms of the sensing interferometer, performing a linear sweeping frequency shown in Figure 1a. The light from the reflected measurement arm carries the distributed sensing information, passes through a circulator, and then recombines with the reference light. The polarization controller and polarization beam splitter split the light in half between two orthogonal polarization states into two detectors, S and P. An inverse Fourier transform is then applied to the gage length, and two peaks are observed from the cross-correlation of spectra between the baseline and stimulated states [18,26].

The distributed sensing measurements performed by the interrogator influence the interference patterns measured by the optical detectors where the physical location of scattering in the sensing fiber are related to the time of flight of light through the fiber. The inverse Fourier transform corresponds to amplitude and phase in a frequency domain converted to a time domain which can be scaled to units of length using the sensing fiber group index and speed of light [23,27,28]. The spectral shift from cross–correlation is based on the relationship of a baseline reflection spectrum, *U_j_*(*v*), where *j* represents a segment of the optical fiber and *v* is the optical frequency. When the fiber is subjected to a change in strain the fiber segment *j* will undergo a change in reflection spectrum, Δ*u_j_* and can be represented as *U*_j_(*v*-Δ*v*_j_). A cross-correlation operation is performed on *U_j_*(*v*) and *U*_j_(*v*-Δ*v*_j_) to calculate the reflection spectrum shift, Δ*u_j_*, which can be related to a resonance wavelength shift, Δ*λ* of a Bragg grating. The relationship is analogous to Bragg grating and can be represented in the following form:

(1) $$\frac{\Delta \lambda }{\lambda }\;=\;\frac{\Delta v}{v}\;=\;K_{\varepsilon }\varepsilon \;+\;K_{T}T$$

where *λ* is the mean optical wavelength, *v* is the frequency, ε is the measured strain, and *T* is the measured temperature. The coefficient *K_ε_* is a strain constant and *K_T_* is a temperature calibration constant to relate spectral shift to a strain or change in temperature values. The strain can be related to Equation (1) in the absence of temperature change:

(2) $$\varepsilon \;=\;-\frac{\bar{\lambda }}{cK_{\varepsilon }}\Delta v$$

where $\bar{\lambda }$ is the center wavelength of the scan and *c* is the speed of light. Similarly, the temperature, Δ*T*, change can be represented using in the following relationship in the absence of the strain:

(3) $$\Delta T\;=\;-\frac{\bar{\lambda }}{cK_{T}}\Delta v$$

where Δ*T* is the change in temperature [23].

**Figure 2:**
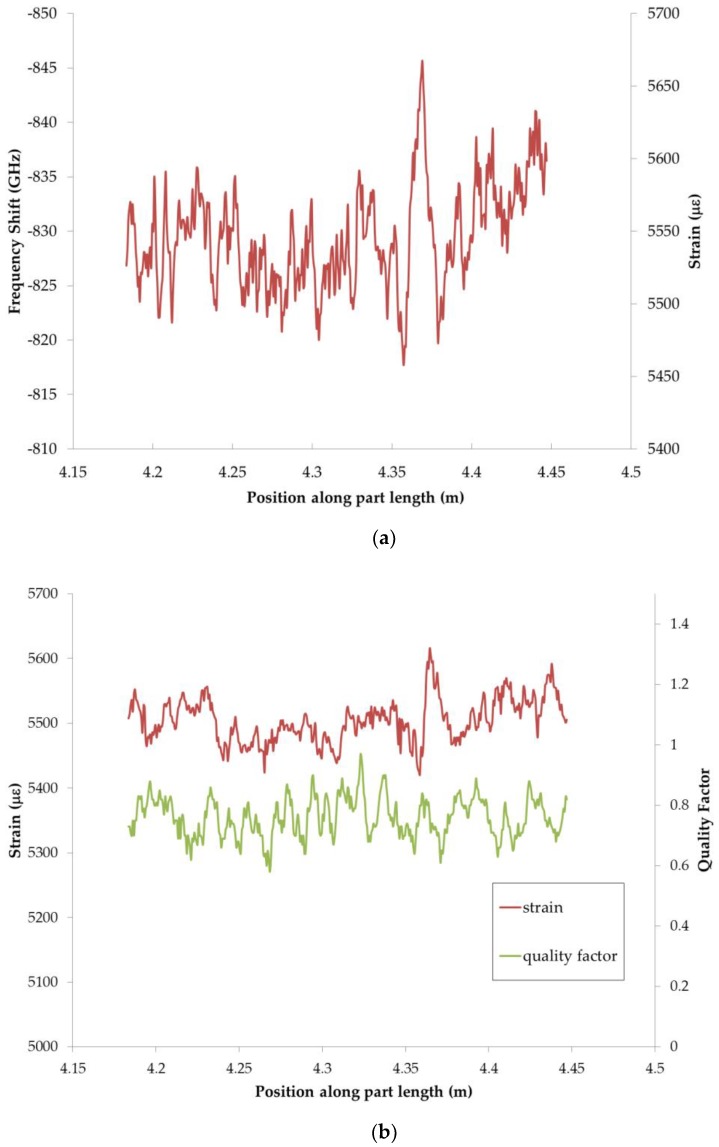
Graphical representations of (**a**) Rayleigh shift measured for section of the fiber sensor and (**b**) relationship of spectral quality factor corresponding to the strain values.

Figure 2a shows example Rayleigh shift curves and corresponding strain converted using HD-FOS. An example quality factor curve for the same strain measurement from Figure 2a is shown in Figure 2b. According to LUNA, the correlation is based on a quality factor of the spectral shift between the measurement and a referenced measurement. Quality factor is a metric that describes the strength of the cross correlation used to determine spectral shift for a single gage. This has a range of 0 to 1 where 1 is a perfect correlation, and 0 is no correlation. For example, calculation using the ODiSI-B unit is used where the data is normalized to identify the strong peak within the correlation results where a value of 1 corresponded to a strong correlation and a value of 0 to a weak correlation. The peak height corresponds to the quality of correlation and the location corresponds to the spectral shift. Note that the quality factor above the noise floor 0.2 to 0.3 range [23]. Figure 2b shows that the quality factor meets the correlation threshold to sufficiently measure the strains.

The sensors are single mode made of used fused silica coated with polyimide exhibiting high mechanical properties with an intrinsic strength of approximately 14 GPa. The fibers are “proof” tested to 690 MPa corresponding to a measurable strain range of +/−50,000 με [29]. The HD-FOS used with ODiSI-B in this work utilized APC connectors and termination with the ability to measure +/−10,000 με, corresponding to a sweep range of 40 nm. Similarly the fiber sensors used with an ODiSI 6100 unit have the ability to measure +/−12,000 με [22,23]. Note, these are conservative strain range measurements used for both interrogators are well within the mechanical strength boundaries indicating the sensors are suitable to be subjected to various mechanical and temperature loadings in harsh environmental conditions.

## 3. Coefficients of Thermal Expansion Measurements

The coefficients of thermal expansion are obtained by measuring strains as a function of temperature and determining the slopes of the thermal strain versus temperature curves. The thermal behavior of a thin (2-dimensional) unidirectional composite (reinforcing fibers aligned in one direction) lamina is fully characterized in terms of two principal coefficients of thermal expansion (CTE), α_1_ and α_2_ along the fiber and transverse directions. After determining the principal coefficients α_1_ and α_2_, the coefficients referred to any system of coordinates x, y are obtained by transformation relations for varying fiber orientation. For chopped carbon fiber composite, the unidirectional lamina are varying the fiber direction spatially and through thickness, and such transformations in three-dimensions is too complex to formulate. However, with HD-FOS, we directly measure the CTE along the direction of the optical fiber and reflects the through thickness response, a great advantage for using fiber-optic sensing for such complex measurement applications in chopped fiber composites. The CTE is a critical material property to monitor for the design and end use application when assembling dissimilar materials. A small CTE value, α_1_, in the longitudinal direction of the fiber is observed compared to CTE in the transverse direction, α_2_, of the fiber when undergoing an increased change in temperature. Conversely, ideally an isotropic thermal expansion is observed for the same change in temperature increase for the epoxy matrix. The CFSMC composite material, consisting of chopped carbon fibers impregnated with epoxy resin matrix, where it is well known that it has a mismatch between fiber and matrix, requires further consideration to obtain accurate CTE measurements of the composite due to its complex microstructure. Additionally, the CTE of carbon fiber and SMC are temperature dependent, where the temperature can change the mechanical properties [30,31]. Conventional techniques including strain gages, dilatometery, and digital image correlation (DIC) have been utilized to measure CTE [32,33,34]. FBG sensors have been used for CTE measurements subjected to thermal cycling [32]. However, due to the FBG sensors are intrinsic point sensors which can consist of multiple inscribed gratings and multiplexing along the sensor, achieving distributed high spatial resolution remains a challenge [35]. OFDR using RBS can resolve the spectral shift to obtain a strain profile of the local variations through each gage segment, *U_j_*(*v*) of the optical fiber. This can be obtained by relating the changes in strain or shift in spectrum along the fiber from a referenced state. Using the relationship defined in Equation (1), the coefficient of linear thermal expansion (CLTE) is determined using:

(4) $$\frac{\;\Delta \lambda_{c}}{\lambda_{c}}\;=\;\frac{\Delta v_{c}}{v_{c}}\;=\;\alpha \Delta T_{c}$$

where α is the coefficient of thermal expansion, λ_c_ is the mean optical wavelength, *v_c_* is the frequency, and Δ*T_c_* is the homogenous temperature change within the oven measured using thermocouples.

## 4. Materials and Methods

### 4.1. Carbon Fiber-Reinforced Composite and Aluminum Hybrid Joint Using Epoxy Adhesives

Two epoxy-based adhesives (EAs), namely 1K EA and 2K EA supplied by Dow Chemical company, were selected to evaluate strain development and residual strain response while bonding dissimilar materials, CFSMC and Al panels.

The mechanical properties of epoxies and adherends are summarized in Table 1 and Table 2, where 1K EA has a significantly higher modulus or stiffness than 2K EA, however, 2K EA can sustain a greater amount of strain to failure (tensile strain or deformation prior to failure) compared to the 1K EA.

### 4.2. Thermal Digital Image Correlation (TDIC) and High-Definition Fiber-Optic Sensor (HD-FOS) Benchmarking

**Figure 3:**
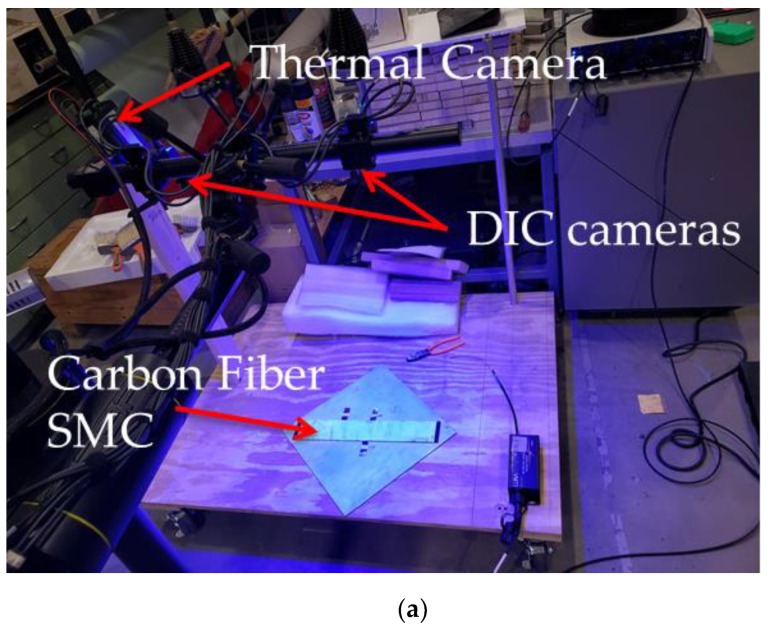

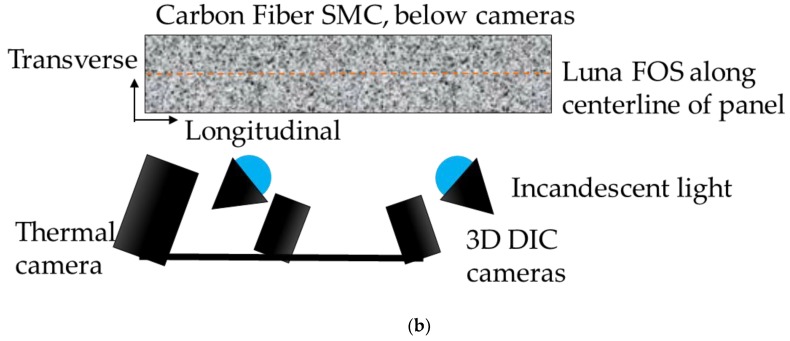
Image (**a**,**b**) schematic of the experimental setup for thermal digital image correlation (TDIC) coupled with the high-definition fiber-optic sensor (HD-FOS).

To validate the accuracy of the HD-FOS–based strain measurement for discontinuous carbon fiber–reinforced composite material based hybrid joints, an isothermal calibration experiment was conducted using TDIC. A 1 m-long HD-FOS, 155 microns in outer diameter, was mounted along the centerline of a composite panel with approximately 457 mm length × 76 mm width × 2.6 mm depth using a cyanoacrylate-based adhesive (M-bond 200, Micro-Measurements), as illustrated in Figure 3. The goal of this study was to measure strain response at elevated temperatures, collecting data at 31.3 Hz with a gage length of 1.3 mm (gage pitch 0.65 mm) using the Optical Distributed Sensor Interrogator (ODiSI) 6100 [36]. For TDIC measurements, an infrared (IR) camera was coupled with a stereo camera 3D digital-imaging correlation (DIC) system, a custom digital-imaging technique to track on-surface strain where the two-camera system allowed evaluation of the local fiber orientation from the surface of the CFSMC panel. The technical details of the equipment setup are summarized in Table 3 and Table 4. The IR camera was an A655sc model from FLIR^®^ Systems, Inc (Wilsonville, OR, USA). The IR camera was calibrated with the measurement temperature range optimized for 40 °C to 150 °C. The emissivity of the material was 0.95. A stereo Aramis 12 megapixel optical camera with an adjustable base was used for DIC measurements, with the IR camera mounted outside and approximately parallel to the left optical camera of the crossbeam supporting the optical cameras.

After curing the M-bond 200 adhesive to attach the fiber-optic sensor, the CFSMC panel was painted matte white and speckled black. A reference image was obtained at room temperature with both camera systems and prior to placing the sample in a 90 °C oven (Fisher Scientific 516G, Hampton, NH, USA) for 30 min to achieve isothermal conditions. A trigger element driven by the Aramis software allowed both camera systems to capture images simultaneously at rates up to 1 Hz. After heating, the sample was returned to the reference viewing location and imaged at 1 Hz with both camera systems as the panel cooled to room temperature. A series of scripts in the Aramis software mapped the images and pixel data from the IR camera to the DIC images to inspect spatially resolved, thermally induced strains on the part surface. The IR camera had a resolution of 640 × 480 pixels compared to the 4096 × 3000–pixel resolution of the Aramis cameras. However, by spatially matching the view from the IR camera to the left view of the Aramis cameras, the mapping scripts were able to match the temperature across the surfaces of the higher-resolution optical images. Time stamps were compared between the ODiSI 6100 data files and the TDIC images to match the correct files for the cooling period.

A matrix of data points was exported from the DIC software at 1 mm spacing along two lines parallel to the HD-FOS, approximately 2 mm above and below the centerline. The slight displacement was set to ensure the data were extracted along the lines for comparison to avoid introducing any inaccurate measurements of the HD-FOS sensor and the adhesive holding it to the surface. A custom-developed script in MATLAB was created to compare quantitatively the longitudinal strain along the DIC comparison lines and HD-FOS at the target temperature. Due to slight displacement between TDIC and the HD-FOS data was interpolated to align with TDIC quantitatively using a custom Python script.

**Figure 4:**
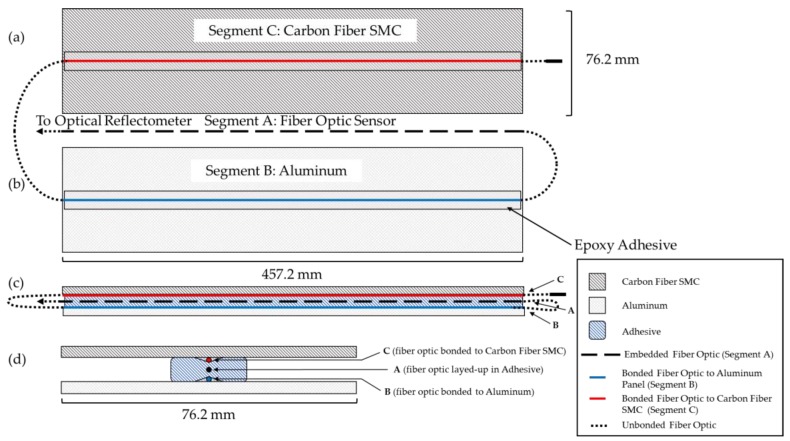
Schematic of (**a**) carbon fiber SMC (CFSMC) and (**b**) aluminum (Al) panel with a HD-FOS; (**c**,**d**) cross sections of sandwich specimens bonded to measure strain response to temperature. A carbon fiber SMC panel (Segment C) is joined to an Al panel (Segment B) with a HD-FOS (Segment A) embedded in an epoxy adhesive.

**Figure 5:**
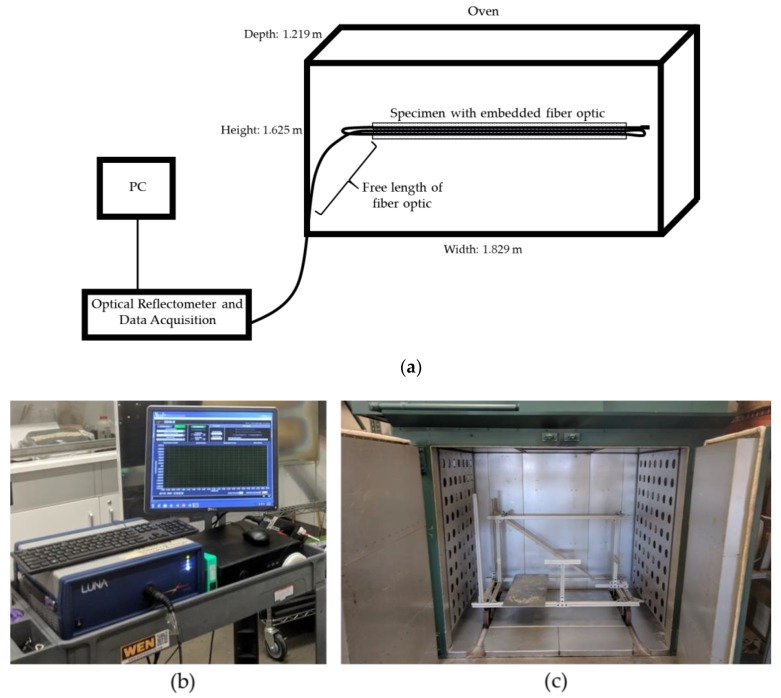

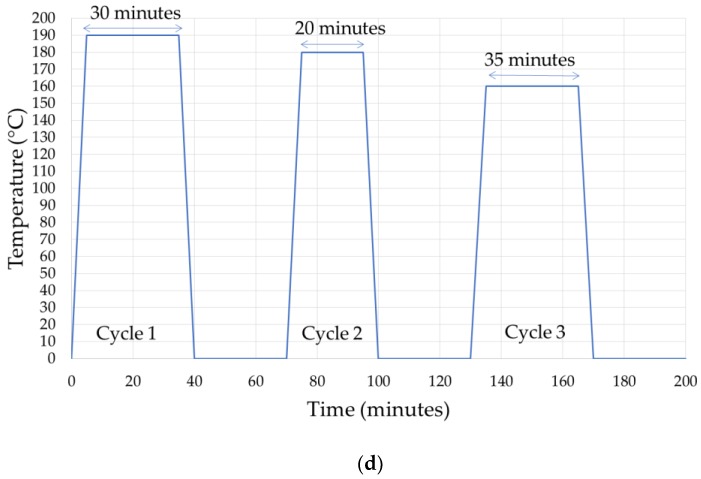
(**a**) Schematic and (**b**,**c**) photographs of the experimental setup for strain measurement during the thermal cycle. (**d**) Graphical representation of the thermal cycle after post curing of the carbon fiber SMC (CFSMC) and aluminum (Al) adherends to simulate electrodeposition paint (E-coat) process.

Figure 4 shows the schematic of a hybrid composite–to–metal joint, where a CFSMC panel (457.2 mm length × 76.2 mm width × 2.6 mm thick) and Al panel (457.2 mm length × 76.2 mm width × 0.9 mm thick) were joined with HD-FOS embedded in the adhesive. One side of each panel was lightly sanded using 220-grit sand paper to ensure sufficient bonding of the HD-FOS to the surfaces. The panels were then wiped down with ethanol and a paper towel to remove any debris. A 5 m HD-FOS was mounted using M-bond 600 (Micro-Measurements) adhesive along the critical segments shown in Figure 4 where segment A corresponded to the free length of the embedded sensor in epoxy adhesive, segment B was attached to the Al panel, and segment C was attached to the CFSMC panel. For segment B and segment C, the HD-FOS was mounted uniaxially along the centerline of the panel. The ODiSI-B interrogator shown in Figure 5a,b was used to measure and compare strain development from fiber-optic sensors. Prior to thermal cycling, to ensure that the M-Bond 600 was sufficiently cross-linked and confirm the attachment of HD-FOS to the panel surface, cure and post-cure steps were required. The CFSMC and Al panels without 1K or 2K epoxy adhesives were placed in an oven shown in Figure 5c and cured at 140 °C for 40 min. The panels were then removed from the oven and allowed to cool to room temperature. To obtain a reference baseline, a tare for the HD-FOS was performed and the M-bond 600 was then post-cured at 190 °C for 30 min. Saint-Gobain Teflon tape was added along each panel’s 457.2 mm length as a spacer to provide an approximately 0.25 mm bond gap for the embedment of the HD-FOS between the two adherends. For each of the two CFSMC/Al smart joints, the adhesive was then added along the length of the HD-FOS on the CFSMC panel to achieve approximately 12.7 mm in width when compressed between the panels. The free length (segment A) of the HD-FOS was laid in the bead of adhesive and a toothpick was used to bury the optical fiber in the adhesive, and all the while slight tension was applied at both ends to keep the optical fiber straight. The Al panel was mechanically joined to the CFSMC panel with adhesive, with the embedded HD-FOS between the two adherends, via 10 steel binder clamps applying a compressive load along the edge of the sample to ensure a proper bond gap.

### 4.3. Thermal Cycling to Simulate the Automotive Paint Process

The electrophoretic painting process (E-Coat) process is a cross between plating and painting. It is a process where a metal part is immersed in a water-based solution containing a paint emulsion. An electric voltage is applied to the part causing the paint emulsion to condense onto the part. A thermal cycle program was performed for the CFSMC/Al smart joint to mimic the electrodeposition of paint (E-coating). Figure 5d shows a graphical representation thermal cycle used in this study. The oven was set to 190 °C to mimic the first step in an automotive paint cycle. The HD-FOS was tared to obtain a baseline reference. The CFSMC/Al smart joint, joined panels, sensor, and clamp were placed in the oven for 30 min, removed from the oven, and allowed to cool to a room temperature of approximately 30 °C. The oven was then set to 180 °C, the HD-FOS recorded the strain values for 20 min, and the hybrid joint was again removed and allowed to cool to room temperature. To mimic the last step in E-coat paint process, the oven was set to 160 °C, and the hybrid joint assembly was placed in the oven for 35 min and allowed to cool at room temperature. The strain data was acquired at 0.65 mm mean gage pitch intervals along the entire 5 m length of the fiber-optic sensor.

## 5. Results and Discussion

Due to the complex behavior of the internal strain and residual strain development of the CFSMC/Al smart joint subjected to various thermal cycles, the authors proposed the following experimental program:

(1) Measurement of strain for a CFSMC specimen in an unstressed state over a specified physical length using thermal digital image correlation (TDIC) optical method and HD-FOS using OFDR signal to compare the resulting measurement for both techniques.
(2) Monitor the strain evolution and residual strains of CFSMC/Al smart joint subjected to three thermal cycles from the OFDR signal obtained from the embedded HD-FOS.
(3) Calculate the CLTE using the relationship previously described in equation 4 to determine the thermal expansion along the CFSMC/Al smart joint based on the local strain Δε, derived from the proportional relationship between the distribution strain and spectral shift [19].

### 5.1. High-Definition Fiber-Optic Sensor Results Evaluation Using Thermal Digital Image Correlation Technique for Carbon Fiber Sheet Molding Compound-Based Composite Panel

**Figure 6:**
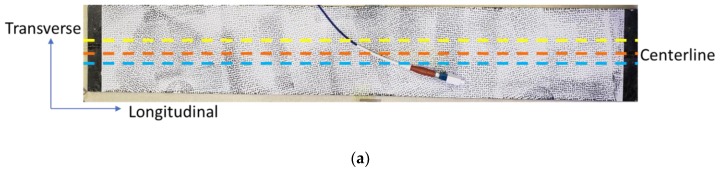

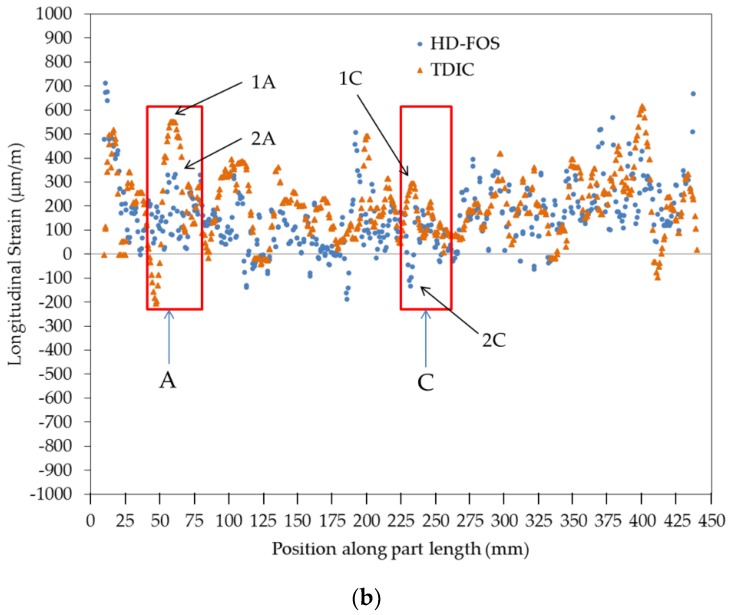
(**a**) Image of the CFSMC panel with fiber-optic sensor (FOS) mounted along the centerline and a speckle pattern on the surface for TDIC measurement. Note the yellow and blue lines are reference lines from TDIC technique used to the compare strain measurements to the HD-FOS strain measurements in the centerline region (orange). (**b**) Comparison of residual strains as a function of location measured by the fiber-optic sensor versus the TDIC technique.

The strain values are governed by spectral shift measurements output from the ODiSI interrogator units, using a polynomial to express the following relationship:

ε(*v*) = A_1_*d* + A_2_*d*^2^ (5)

where ε is the strain (μm/m) and d is the spectral shift (GHz). The coefficients A_1_ (με/GHz) and A_2_ (με/GHz^2^) relate ε and d shown in Equation (1) [22,23]. Table 5 shows the factor coefficients values used to convert spectral frequency shift to strain values for the experimental procedures throughout this study. Note the negative coefficient values correspond to an increased strain similar Figure 1d and Figure 2a [37]. Figure 5a shows a picture of the CFSMC panel in an unstressed state with an HD-FOS mounted in the middle, represented by the orange dashed centerline. The surface of this panel was speckle-patterned to conduct TDIC measurements in parallel as described earlier to compare the strain evolution of the panel compared to the HD-FOS during the cooling phase after the panel was heated to 90 °C in an oven and subsequently removed to observe strain measurements comparison between the two techniques. The two lines (yellow and blue) approximately 1 to 2 mm above and below the HD-FOS centerline (orange) mark comparative reference locations for measurements obtained using TDIC. Figure 6b shows the comparative strain measurements along a longitudinal direction measured by the fiber-optic sensor versus TDIC. The data demonstrates the complexity of strain, and thus stress evolution, in a platelet-based CFSMC composite panel. Since the panel is made of platelets of unidirectional carbon fiber infused with resin that undergo significant spreading in an in-plane direction and bending in the through thickness direction, the final microstructure is very complex and varies as a function of location along the fiber-optic sensor.

**Figure 7:**
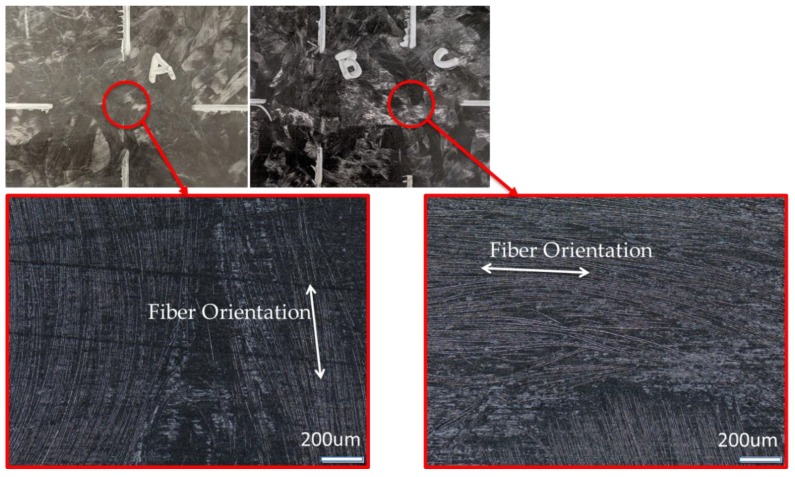
Local fiber orientation effects on thermal expansion of CFSMC (regions A and C from Figure 5b).

For those regions that are dominated by resin expansion, where carbon fiber is perpendicular to the longitudinal direction, large thermal expansion strains were expected. However, for those regions in the composite panel where carbon fiber is largely oriented along the longitudinal direction identified in Figure 6a, negligible thermal expansion was expected. This is attributed to the carbon fiber contracting when heated due to its turbostratic graphite crystal microstructure along the fiber axis. A difference of approximately 217 μm/m between peak locations 1A and 2A was observed for region A between TDIC and HD-FOS around the 57 to 61 mm HD-FOS position for the peak strain positions along the fiber optic. Similarly, a difference of approximately 426 μm/m was observed between 1C and 2C for region C around the 232 to 233 mm HD-FOS position along the fiber optic. The positions outside the regions A and C exhibited strong overlap between the TDIC and HD-FOS with the exception of edge position, near 0 mm and 450 mm. This can be attributed to the localized fiber orientation due to constraints from the molded part. The difference in the spectra is due to the HD-FOS strain measurement based on an averaging function. Utilizing 12 MP Aramis stereo cameras parameters, described in Table 3, the strain response of the surface of the panel is captured where a centerline of data points is extracted and compared to the HD-FOS used in this study. The field of view was set to capture the full length of the panel (457.2 mm) and resulted in an image resolution of 0.14 mm/pixel. A fine speckle pattern was used to track the surface per manufacturer recommendations for the field of view and the subset (facet) size used for data analysis was 24 pixels (3.36 mm) with 12 pixels (1.68 mm) separation between the center points of the subset as indicated. The data for each subset is assigned to the subset center point. Considering both a subset overlap and a 3D surface analysis, the points on the extracted centerline region of the surface analysis were between 0.9 and 0.5 mm apart. Comparatively, the HD-FOS data points were separated by 0.65 mm, uniformly. Thus, the subset size from the cameras gave a similar order of magnitude in data point separation, but the slight difference that remains would mean slightly different regional averaging for the two sensors and thus the discrepancies in output strain data seen in Figure 6b. Although there are slight differences between the strain measurements, the similar magnitude of data point spacing between the two techniques capture similar behavior in higher strain regions as well as along the length of the CFSMC part (Figure 6a) shown in Figure 6b. The fiber-optic sensor correctly captured all these complex microstructure effects, as shown in Figure 6b, with good agreement with results from the TDIC. Figure 7 shows the microstructure in detail, corresponding to two regions identified as A and C in Figure 6b. Both low-resolution photographs and high-resolution optical micrographs are shown in Figure 7, demonstrating that the local fiber orientation effects on thermally induced expansion strains were correctly captured by the fiber-optic sensors. TDIC is more sensitive spatially to the surface of the panel, however, this strain measurement correlation with the HD-FOS provides an unambiguous validation of adhering the HD-FOS directly to the CFSMC, measuring actual strain without requiring any additional correction factors associated with HD-FOS mounting techniques.

### 5.2. Carbon Fiber-Reinforced Sheet-Molded Composite (CFSMC)-Al Smart-Joint Response Resulting from Thermal Cycling

The spatial local strain variations of CFSMC/Al smart joints were evaluated using HD-FOS in response the E-coat process. This included evaluation of (1) local material and geometric effects (2) displacement of adherends and epoxy adhesive to substrates, and (3) spatially resolved coefficient of thermal expansion as measured by the HD-FOS.

#### 5.2.1. Displacement Measurements

**Figure 8:**
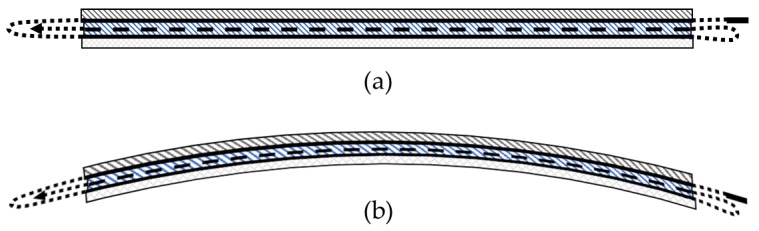
Schematic showing the state of the uncured adhesive in a hybrid joint (**a**) and post-cured state of the adhesively joined assembly (**b**).

**Figure 9:**
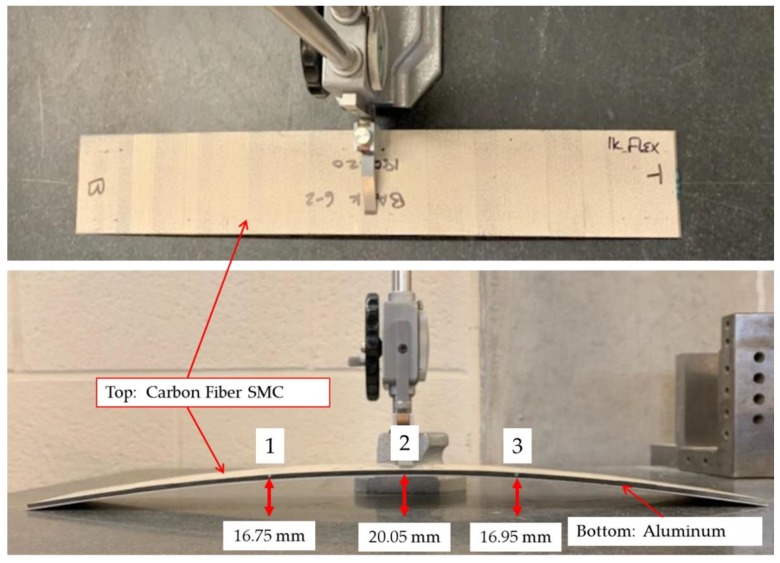
CFSMC/Al smart joint bonded with 1K epoxy adhesive (EA) with a 0.25 mm bond gap, after thermal cycling.

Figure 8 and Figure 9 show warpage from the thermal cycling of the CFSMC/Al smart joint, demonstrating the challenge of joining such dissimilar materials. This warpage can be attributed to the CLTE mismatch between the adherends (CFSMC and Al) and the adhesive. CFSMC is subject to internal strains induced by the cure shrinkage of the epoxy [38]. Furthermore, Table 6 shows the mean displacement measured for all four samples; sub-millimeter displacement was detected by HD-FOS for each thermal cycle, confirming the displacement of the increase (or materials thermally relaxed) for elevated temperatures. The HD-FOS, embedded in the adhesive accordingly, measured the 2K EA as having a greater displacement than the 1K EA, primarily because the 2K EA has a lower modulus. Conversely, the HD-FOS measured a higher displacement in the CFSMC and Al for the 1K EA than the 2K EA, primarily due to the stiffer 1K EA inducing a higher displacement of the adherends. The ability to precisely measure these values quantitatively and their distribution spatially is very unique to the high density and distributed fiber optic-based sensing technology.

#### 5.2.2. Strain, Residual Strain, and Coefficient of Linear Thermal Expansion Measurements

**Figure 10:**
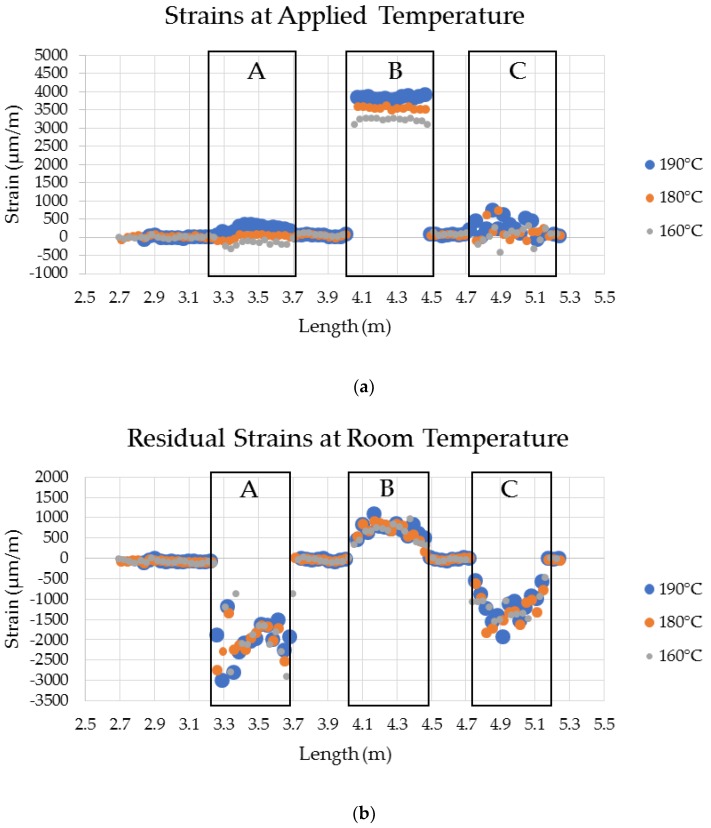
(**a**) Experimental and (**b**) residual strains for the CFSMC/Al smart joint bonded with 1K EA with a 0.25 mm bond gap, where section A is a fiber-optic sensor, section B is an Al panel, and section C is a CFSMC panel.

Figure 10a,b show the clear development of strain, and the subsequent residual state of strain after cooling back to room temperature for each thermal cycle excursion during the E-coat process. The data generated by the HD-FOS for the CFSMC/Al smart joint for thermal cycle is representative of all three material systems where segment A is the segment of the sensor embedded in the adhesive, segment B is adhered to the Al panel, and segment C is adhered to the CFSMC panel. Very large compressive residual strains in CFSMC and tensile residual strains in Al were seen after the end of the simulated E-coat process. The adhesive strain values in Region A is relatively close to zero due to the thermal coefficient in the longitudinal direction does not significantly change at the elevated temperatures. CFSMC shows localized strain variation with higher strains at 190 °C and 180 °C. However, at 160 °C it is clear the CFSMC the strain is close to zero. The primary reason for this behavior is due to CFSMC and Al are constrained by the stiffness of the adhesive. Hence, the CFSMC is subjected to compression strains and Al is subjected to tensile strains induced from residual strains shown in Figure 8b and Figure 9 [5]. Although, the general trends of strain developments can be observed in Figure 10, a closer investigation was warranted to view localized strain behavior the CFSMC and Al adherends compared to the epoxy adhesives.

**Figure 11:**
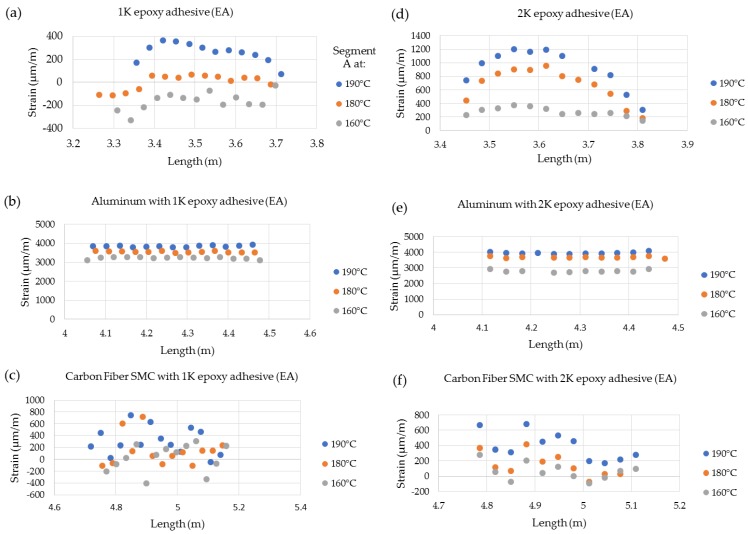
Fiber optic-based expansion strains for a 0.25 mm bond gap (adhesive layer thickness) at maximum temperature during thermal cycles for (**a**) 1K EA, (**b**) Al with 1K EA, and (**c**) CFSMC with 1K EA and (**d**) 2K EA, (**e**) Al with 2K EA, and (**f**) CFSMC with 2K EA.

**Figure 12:**
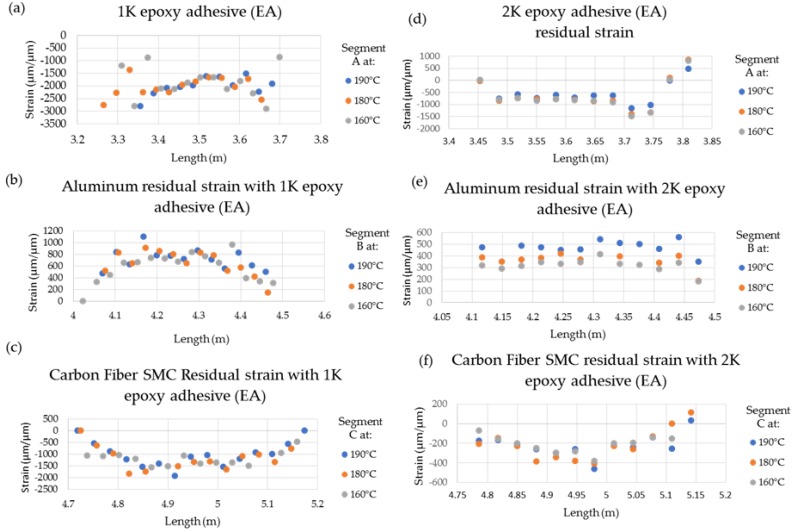
Residual strains after reaching room temperature with a 0.25 mm adhesive bond gap thickness for (**a**) 1K EA, (**b**) Al with 1K EA, and (**c**) CFSMC with 1K EA and (**d**) 2K EA, (**e**) Al with 2K EA, and (**f**) CFSMC with 2K EA.

Figure 11 shows the spatial strain variation along the CFSMC/Al smart joint for each stage of the thermal cycle, where a clear increase in the strain is shown for the two adhesives. The Al adherends for both 1K EA (Figure 11b) and 2K EA (Figure 11e) showed similar relatively uniform strain behavior and strain values for each thermal cycle. The Al strain for 1K EA was 3839 ± 21 με (190 °C), 3536 ± 19 με (180 °C), and 3212 ± 29 με (160 °C). Similarly, the Al strain values for 2K EA was 3941 ± 35 με (190 °C), 3663 ± 29με (180 °C), and 2777 ± 44 με (160 °C). The strain value (3839 ± 21 με) for Al used with the 1K EA was slightly lower (2.6%) than the aluminum (3941 ± 35 με) used with 2K EA at 190 °C. The strain values overall decreased at temperatures 190 °C to 160 °C for both Al and the epoxy adhesives was approximately 20% compared to for aluminum used with 1K EA compared to approximately 42% reduced mean strain for aluminum used for 2K EA. The effect of adhesive modulus properties on the evolution of strain for each of the three material systems, as well as the subsequent residual strain, is clearly evident from the data shown in Figure 12. The 2K EA resulted in significantly less residual strain in both CFSMC and Al, thus performing better than 1K EA by minimizing CTE mismatch–induced thermal distortions.

**Figure 13:**
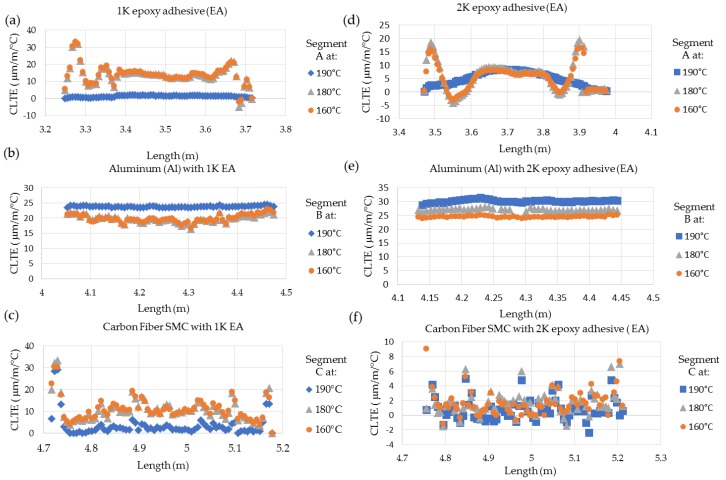
Variation of coefficient of linear thermal expansion (CLTE) with 0.25 mm adhesive bond gap thickness for (**a**) 1K EA, (**b**) Al with 1K EA, and (**c**) carbon fiber SMC (CFSMC) 1K EA and (**d**) 2K EA, (**e**) Al with 2K EA, and (**f**) CFSMC with 2K EA.

Figure 13 and Table 7 show the coefficient linear thermal expansion (CLTE) profile and mean CLTE values interpreted from the HD-FOS, allowing comparison to the thermal responses of the CFSMC/Al smart joints for a progressive thermal cycle. Significant differences in CLTE were observed, as expected, for the adherends and epoxies for each smart joint. The Al CLTE profiles are relatively uniform for 1K EA (Figure 13b) and 2K EA (Figure 13e). The CLTE for Al bonded with 1K EA was 22 ± 0.42 μm/m/°C (190 °C) and 18 ± 0.48 μm/m/°C at 180 °C and 160 °C. The CLTE for Al bonded with 2K EA was 30 ± 0.21 μm/m/°C (190 °C), 26 ± 0.28 μm/m/°C (180 °C) and 21 ± 0.19 μm/m/°C (160 °C). The CLTE of Al generally increased with higher temperatures, however, due to the Al is bonded to the adhesive, a thermal contraction is observed for both CFSMC/Al smart joints [39]. As shown in Figure 13b, the Al bonded with 1K EA showed local strain variations along the smart joint particularly at 180 °C and 160 °C compared to the Al bonded with 2K EA (Figure 13e) where the strain slightly decreased part for each thermal cycle due to thermal contraction within the CFSMC/Al smart joint. The CLTE for 2K EA was 6 times greater than 1K EA at 190 °C. Conversely, the 1K EA was 1.5 times and 2 times greater than 2K EA at temperatures 180 °C and 160 °C, respectively. The spikes shown for both EAs at 180 °C and 160 °C correspond to large CLTE due to residual strains developing on the extremities of the CFSMC/Al smart joint. The CFSMC CLTE shows a stronger overlap of CLTE for the CFSMC bonded with 2K EA (Figure 13f) compared to CFSMC bonded with 1K EA (Figure 13c) where the CLTE at 190 °C is distinguishable from 160 °C and 180 °C thermal cycles. As shown in Table 7, the CFSMC bonded with 2K EA had a constant CTE mean value of 3 μm/m/°C. Similarly, the mean CLTE value for CFSMC with bonded with 1K EA was 3 μm/m/°C at 190 °C, however, the CLTE was approximately 3.3 and 3.6 times higher than the CFSMC with bonded with 2K EA at 180 °C and 160 °C.

The CLTE CFSMC/Al smart joint with higher modulus 1K EA generally remained constant at temperatures 180 °C and 160 °C, as shown in Figure 13a–c. The CLTE CFSMC/Al smart joint with lower modulus 2K EA was more apt to have similar thermal expansion at each thermal cycle as shown in Figure 13d-f This difference can be attributed to the lower modulus 2K EA not causing as much strain on the CFSMC/Al smart joint adherends compared to the higher modulus 1K EA. Additionally, due to the significant difference in modulus properties between 1K EA and 2K EA shown in Table 1, during the thermal cycle the strain response will behave differently between the adhesives as observed Figure 13a,d. For Figure 13b,e, the combination of carbon fiber composite and adhesive influences the aluminum restricting the thermal expansion due to residual strain development from the composite and adhesive, respectively.

## 6. Conclusions

Evaluation of material joining, especially involving fiber-reinforced composite materials and metals, require spatially distributed sensing using a technology that is least affected by electromagnetic interference, environmental conditions such as corrosion, and does not involve multiple electrical sensor cables such as is necessary with resistive strain gages. In this paper, a novel implementation of high definition fiber-optic sensing based on Rayleigh back scatter signal was demonstrated to monitor in real time the strain development, which can be used to evaluate the structural health of a hybrid material joint for automotive applications. The performance of the ‘SmartJoint’ during a simulated electrophoretic paint process conditions with high spatial resolution was obtained to reveal adhesive join deformation and stress state between carbon fiber composite and aluminum panels bonded using structural epoxy-based adhesives. The distributed fiber-optic sensor successfully provided information on strain evolution with temperature cycling and the resulting residual strain accumulation in response to varying time-temperature loading conditions along the length of the joint. The present paper demonstrates the concept of using such a technique for structural health monitoring in an on-demand sensing mode, providing an exceptionally large density of measurement points with gage lengths as small as 0.65 mm over meters of length. Such data is useful to predict localized strains along an adhesively joined component and its variation with time and temperature and externally applied mechanical stress. This technology has the potential for monitoring aerospace and automotive joints as a function of service state and environmental conditions and shows promise. Results obtained from the fiber-optic sensor were in agreement with the digital-image-correlation-based technique and demonstrated the ability of this method to provide local strain measurements as a function of fiber orientation and matrix-rich regions. This type of information is not possible to measure with existing sensing technologies (for example resistive strain gages) on such large-scale composite or hybrid automotive joints and parts such as lift gates or deck lids. Since the joints are subjected to extreme environments (temperature, humidity, voltage spikes), this sensing technology shows promise for realizing their state-of-health on demand.

## Figures and Tables

**Table 1:** Mechanical properties of adhesives used.

Properties	1K EA	2K EA	Units
Tensile Strength	5	4	MPa
Tensile Modulus	13	3	MPa
Tensile Strain	203	323	%
Lap Shear Strength	12	11	MPa

Note: EA is epoxy adhesive.

**Table 2:** Mechanical properties of composite and aluminum panels.

Properties	Carbon Fiber/Sheet-Molded Composite (SMC)	Aluminum 6061 T6	Units
Tensile Strength	300	310	MPa
Tensile Modulus	40	69	MPa

**Table 3:** Aramis stereo digital image correlation (DIC) cameras.

Parameters	Settings
Technique:	Thermal Digital Image Correlation (TDIC) with IR camera
Cameras:	Aramis AdjusTable 12 MP
Lens:	Schneider Kreuznach APO-XENOPLAN 2.0/24-0005, 24 mm
Sensor/Digitalization	4096 × 3000, 8-bit
Lighting:	Constant lighting, light-emitting diode (LED) lamps
Camera/Imaging Distance:	1000 mm to specimen, 400 mm between cameras
Pixel to mm Conversion:	8
Software:	Aramis Professional 2018
ROI:	457 mm × 76 mm
Subset, Step:	24, 12

**Table 4:** FLIR infrared (IR) camera.

Parameters	Settings
Technique:	Thermal Digital Image Correlation (TDIC) with IR camera
Cameras:	FLIR A655sc
Lens:	FLIR FOL25
Sensor/Digitalization	640 × 480, 16-bit
Emissivity:	0.95
Lighting:	Constant lighting, incandescent bulbs
Imaging Distance:	1000 mm to specimen
Pixel to mm Conversion:	2.1
Software:	FLIR ResearchIR Max

**Table 5:** Calibration coefficients, A_1_ and A_2_, used to convert spectral shift (GHz) values to strain (με) values for the CFSMC and CFSMC/Al with epoxy adhesives smart joints.

Testing Experiment	A_1_ (με/GHz)	A_2_ (με/GHz^2^)	Interrogator Unit
TDIC and HD-FOS comparison	−6.700045109	−0.0000546390	ODiSI 6100
CFSMC/Al with 1K Epoxy Adhesive	−6.688827515	−0.0000451458	ODiSI-B
CFSMC/Al with 2K Epoxy Adhesive	−6.698980808	−0.0000451458	ODiSI-B

**Table 6:** Mean linear displacement of CFSMC/Al smart joint bonded with EAs for various thermal cycles using HD-FOS.

Displacement (mm) at Temperature	1K EA (mm)	Aluminum (Al) with 1K EA (mm)	Carbon Fiber SMC with 1K EA (mm)	2K EA (mm)	Aluminum (Al) with 2K EA (mm)	Carbon Fiber SMC with 2K EA (mm)
190 °C	0.109	1.665	0.259	0.352	1.448	0.130
180 °C	−0.001	1.538	0.168	0.275	1.325	0.039
160 °C	−0.087	1.406	0.084	0.113	1.163	0.022

Note: EA = epoxy adhesive.

**Table 7:** Coefficient of linear thermal expansion properties (μm/m/°C) for a CFSMC/Al smart joint bonded with EAs.

Thermal Expansion Temperature Range (°C)	1K EA (μm/m/°C)	Aluminum (Al) with 1K EA (μm/m/°C)	Carbon Fiber SMC with 1K EA (μm/m/°C)	2K EA (μm/m/°C)	Aluminum (Al) with 2K EA (μm/m/°C)	Carbon Fiber SMC with 2K EA (μm/m/°C)
30–190 °C	1 (1)	22 (6)	3 (5)	6 (3)	30 (4)	3 (2)
30–180 °C	14 (6)	18 (7)	10 (6)	9 (4)	26 (4)	3 (2)
30–160 °C	14 (6)	18 (7)	11 (5)	7 (5)	21 (3)	3 (2)

Note: EA = epoxy adhesive; standard deviation values are in parentheses.
